# Thromboangiitis Obliterans (Buerger's Disease)—Current Practices

**DOI:** 10.1155/2013/156905

**Published:** 2013-09-11

**Authors:** Abhishek Vijayakumar, Rahul Tiwari, Vinod Kumar Prabhuswamy

**Affiliations:** Department of General Surgery Victoria Hospital, Bangalore Medical College and Research Institute, Bangalore 560002, India

## Abstract

Thromboangiitis obliterans (TAO) is a nonatherosclerotic, segmental inflammatory disease that most commonly affects the small and medium-sized arteries and veins in the upper and lower extremities. Cigarette smoking has been implicated as the main etiology of the disease. In eastern parts of the world TAO forms 40–60% of peripheral vascular diseases. Clinical features and angiographic finding are the basis of early diagnosis of TAO. Abstinence from smoking is the only definitive treatment to prevent disease progression. Medical management in form of aspirin, pentoxyfylline, cilostazol, and verapamil increase pain-free walking distance in intermittent claudication, but long term usage fails to prevent disease progression in patients who continue to smoke. Surgical treatment in form of revascularization, lumbar sympathectomy, omentopexy, and Ilizarov techniques help reduce pain and promote healing of trophic changes. Newer treatment modalities like spinal cord stimulation, prostacyclin, bosentan, VEGF, and stem cell therapy have shown promising results. Latest treatment options include peripheral mononuclear stem cell, and adipose tissue derived mononuclear stem cells have been shown to be effective in preventing disease progression, decrease major amputation rates, and improving quality of life.

## 1. Introduction

Thromboangiitis obliterans (TAO) is a nonatherosclerotic, segmental inflammatory disease that most commonly affects the small and medium-sized arteries and veins in the upper and lower extremities. In the characteristic acute-phase lesion, in association with occlusive cellular thrombosis, the acute inflammation involving all layers of the vessel wall led TAO to be classified as a vasculitis. TAO can be distinguished from other types of vasculitis based on its tendency to occur in young male subjects, its close association with tobacco consumption, the rarity of systemic signs and symptoms, a highly cellular thrombus with relative sparing of the blood vessel wall, and the absence of elevated acute-phase reactants and of immunological markers.

Thromboangiitis obliterans (TAO) was first described in 1879, when Felix von Winiwarter, an Austrian surgeon who was an associate of Theodor Billroth, reported in the German Archives of Clinical Surgery a single case of what he described as presenile spontaneous gangrene [1]. Buerger related the cellular nature of arterial thrombosis, as had von Winiwarter, and described the absence of large vessel involvement. It was Buerger who named the disorder “thromboangiitis obliterans”, and only briefly mentioned its relationship with smoking. In 1924, Buerger reported that tobacco use was probably a predisposing factor [2]. 

## 2. Epidemiology 

The prevalence of the disease among all patients with peripheral arterial disease ranges from values as low as 0.5 to 5.6% in Western Europe to values as high as 45 to 63% in India, 16 to 66% in Korea and Japan, and 80% among Jews of Ashkenazi ancestry living in Israel [3]. TAO was initially thought to affect almost exclusively men, since less than 1% of those affected were women. In the most recent studies, the proportion of female TAO patients varies between 11% and 23% [4]. This increase may be due to an increase in smoking among women. 

## 3. Etiology and Pathogenesis

The etiology of Buerger's disease is unknown. Although TAO is a type of vasculitis it is distinct from other vasculitis. Pathologically, the thrombus in TAO is highly cellular, with much less intense cellular activity in the wall of the blood vessel and a preserved internal elastic lamina. In addition, TAO differs from many other types of vasculitis in that the usual immunologic markers—elevation of acute-phase reactants such as erythrocyte sedimentation rate (ESR) and C-reactive protein (CRP), circulating immune complexes, and autoantibodies such as antinuclear antibody, rheumatoid factor, and complement levels are usually normal or negative.

### 3.1. Smoking

Use or exposure to tobacco plays a central role in the initiation and progression of the disease. By using an antigen-sensitive thymidine-incorporation assay, Adar et al. [5] showed that patients with TAO have an increased cellular sensitivity to types I and III collagen compared to that in patients with arteriosclerosis obliterans or healthy males. It is possible that there is an abnormal sensitivity or allergy to some components of tobacco and that this sensitivity in some way leads to an inflammatory small vessel occlusive disease. Purified tobacco glycoprotein (TGP) could be related to changes in vascular reactivity that may occur in cigarette smokers [6]. Matsushita et al. [7] showed a very close relationship between active smoking and an active course of Buerger's disease, using the urine level of cotinine (a metabolite of nicotine) as a measurement of active smoking.

### 3.2. Genetics

There may be a predisposition to development of TAO, although no gene has been identified to date. There is no consistent pattern in HLA haplotypes among patients with Buerger's disease. In the United Kingdom, there was a preponderance of HLA-A9 and HLA-B5 antigens.

### 3.3. Hypercoagulability

Though role of hypercoagulability in pathogenesis of TAO has been proposed, some studies have failed to demonstrate any correlation. Choudhury and colleagues [8] demonstrated that the level of urokinase-plasminogen activator was twofold higher and that of free plasminogen activator inhibitor I was 40% lower in patients with TAO than in healthy volunteers. An increased platelet response to serotonin is described in patients with Buerger's disease [9]. 

Elevated plasma homocysteine has been reported in patients with TAO [10]. This rise may be related to the high prevalence of heavy cigarette smoking or may in some way be directly connected to the disease itself. Patients with TAO who have elevations of homocysteine may also have a higher amputation rate than those who have normal homocysteine levels [11]. There have been several reports of increased anticardiolipin antibody titers in patients with Buerger's disease. Patients with TAO and high antibody titers tend to be younger and to suffer a significantly higher rate of major amputations than those without the antibody [12].

### 3.4. Endothelial Dysfunction

Eichhorn et al. [13] in a study of 28 patient with TAO showed that antiendothelial cell antibody was elevated in 25% of the cases and antiendothelin antibody titers corresponded to disease severity. Makita et al. [14] demonstrated impaired endothelium-dependent vasorelaxation in the peripheral vasculature of patients with Buerger's disease. 

### 3.5. Infection

Initial studies by Buerger and Allen and Brown from Mayo clinic suggested an infectious etiology to TAO linking poor oral hygiene to development of TAO [15, 16]. However, they could not find out any pathogen from the lesions when using the classic orthodox bacterial culture method. Iwai et al. found oral bacteria (periodontal) DNA in the arterial specimens of Buerger disease in 93% of the cases. The oral specimen was the same as that detected in the vessels [17]. The phlebitic lesions of TAO show also oral bacterial DNA by the PCR method [18]. The bacterial transportation system was almost clear in the transmission electron microscopic examination [19]. The platelets engulf the oral bacteria and are carried to terminal vessels within the platelets. An embolic occlusion with platelet aggregated mass including the oral bacteria is one possible explanation of the mechanism of TAO [20]. The genetic relationship was also studied, and the conclusion was that HLA and the CD14 gene polymorphism which lead to defective immunity towards bacterial lipopolysaccharides increases susceptibility to TAO in southeast Asian population [21].

### 3.6. Immunologic Mechanisms

The immune system seems to play a critical role in the etiology of TAO. However, knowledge about immunological aspects involved in the progression of vascular tissue inflammation, and consequently the evolution of this disease, is still limited. TAO may actually be an autoimmune disorder with antibodies directed towards vascular endothelium in response to antigens in tobacco. The presence of different antibodies, such as antinuclear, antielastin, anticollagens I and III, and antinicotine antibodies, as well as identification of deposits of immunoglobulin (Ig) G, IgC3, and IgC4 in the blood vessels of patients, provided evidence to the theory of the immune character of TAO [22]. Maslowski et al. [23] suggested that anticardiolipin antibodies are important for the pathogenesis of TAO. However, patients with generalized periodontitis had significantly greater titres of IgG or IgM anticardiolipin antibodies, and these levels were significantly higher in smokers than in nonsmokers. In addition, elevated titres of IgG against periodontal pathogens are related to development of TAO [24]. In a study by Halacheva et al. [25] biopsy specimen of arteries of TAO patients were studied with immunohistochemistry for presence of tumor necrosis factor-*α* (TNF-*α*) and expression of intercellular adhesion molecule-1 (ICAM-1), VCAM-1, and E-selectin. It was seen that endothelial cells are activated in TAO, and that vascular lesions are associated with TNF-*α* secretion by tissue-infiltrating inflammatory cells, ICAM-1, VCAM-1, and E-selectin expression on endothelial cells and leukocyte adhesion. 

## 4. Pathology 

*Acute-phase* lesion is characterized by acute inflammation involving all coats of the vessel wall, especially of the veins, in association with occlusive thrombosis. Around the periphery of the thrombus, there are often polymorphonuclear leukocytes with karyorrhexis, the so-called microabscesses.

*Intermediate phase* in which there is progressive organization of the occlusive thrombus in the arteries and veins. At this stage, there is often a prominent inflammatory cell infiltrate within the thrombus and much less inflammation in the vessel wall. 

*Chronic phase* or end-stage lesion is characterized by organization of the occlusive thrombus with extensive recanalization, prominent vascularization of the media, and adventitial and perivascular fibrosis.

In all three phases, the normal architecture of the vessel wall subjacent to the occlusive thrombus and including the internal elastic lamina remains essentially intact. These findings distinguish TAO from arteriosclerosis and from other systemic vasculitides, in which there is usually more striking disruption of the internal elastic lamina, and the media, disproportional to the disruptions attributable to aging change. Buerger's disease is segmental in distribution; “skip” areas of normal vessel between diseased segments are common, and the intensity of the periadventitial reaction may be quite variable in different segments of the same vessel. 

## 5. Clinical Features

Classic presentation of Buerger's disease is in a young male smoker with the onset of symptoms before the age of 40 to 45 years. TAO manifests as migratory thrombophlebitis or signs of arterial insufficiency in the extremities. In a study by Sasaki et al. [26] the subjects were 749 men and 76 women, with a mean age of 50.8 ± 0.4 years. In 42 patients (5.1%) involvement was limited to upper extremity arteries, in 616 patients (74.7%) disease was limited to the lower extremity, 167 patients (20.2%) showed involvement of both upper and lower extremities. The most frequently affected arteries were the anterior (41.4%) or posterior (40.4%) tibial arteries in the lower extremities and the ulnar artery (11.5%) in the upper extremities. In this report, approximately 25% of the patients had upper extremity involvement.

Two or more limbs are almost always involved in Buerger's disease. In the series reported by Shionoya and Rutherford [27], two limbs were affected in 16% of patients, three limbs in 41%, and all four limbs in 43% of patients. In a study by Barlas et al. [28] patients with TAO (2468 total; 94.5% men and 5.5% women) at the time of admission, 78.2% had rest pain, 58% had intermittent claudication, 17.6% had supericial thrombophlebitis, 10.5% had Raynaud's phenomenon, and 68.9% had ischemic ulcers. A staging system for clinical symptoms was proposed by Rutherford as described in Table 1. A clinico-pathological classification was proposed by Lerich et al. and later modified by Fontaine as described in Table 2. 

### 5.1. Extremity Involvement

Claudication in the arch of the foot is an early sign and is suggestive of, or even specific to, TAO. This condition is a manifestation of infrapopliteal vessel occlusive disease. As the disease progresses, typical calf claudication and eventually ischemic pain at rest and ischemic ulcerations on the toes, feet, or fingers may develop. Ischemia of the upper limbs is clinically evident in 40–50% of patients, but may be detected in 63% of patients by Allen's test [29] and in 91% of patients by arteriogram of the hand and forearm [30]. In the Allen's test, the examiner places the thumbs to occlude the radial and ulnar arteries of one hand of the patient. The patient opens the fist and the examiner then releases pressure from the radial artery but not the ulnar artery. If the radial artery distal to the wrist is patent, there is prompt return to colour to the hand (negative test). If the artery is occluded, the hand will remain pale (positive test). The maneuver is repeated with the pressure released from the ulnar artery but not the radial artery. 

### 5.2. Superficial Thrombophlebitis

Superficial thrombophlebitis is observed in 40–60% of cases. Deep vein thrombophlebitis is unusual and suggestive of an alternative diagnosis, such as Behcet's disease. This superficial thrombophlebitis is migratory and recurrent and affects the arms and legs. Migrating phlebitis (phlebitis saltans) in young patients is therefore highly suggestive of TAO [31].

### 5.3. Systemic Signs and Symptoms

Systemic signs and symptoms are very rare in patients with TAO. There has been occasional reports involvement of visceral vessels. Digestive ischemia may manifest as abdominal pain, diarrhea, weight loss, or melena. Intestinal perforation and mesenteric infarction may occur. There have been reports of TAO initially presenting as small bowel ischemia and colonic obstruction [32, 33].

In some of these cases, visceral artery damage results more likely from atherosclerosis, favored by or associated with TAO. Thus, when TAO occurs in an unusual location, diagnosis should be made only after the identification of typical inflammatory vascular lesions on histopathological examinations.

 Central nervous system involvement has been reported in TAO which may present as transient ischaemic attacks or ischaemic stroke. Postmortem histological examinations have demonstrated inflammation of the small- and medium-sized arteries of the leptomeninges or even of the meninges or veins [34].

Coronary artery involvement is extremely rare [35]. There have been reports of coronary artery involvement presenting as acute myocardial infarction [36].

TAO may present with joint manifestations [37]. On careful history taking about 12.5% may report joint problems before the preocclusive phase [38]. Patients present recurrent episodes of arthritis of the large joints, with transient, migratory single-joint episodes accompanied by local signs of inflammation. The wrists and knees are the most frequently involved joints. The duration of signs and symptoms ranges from 2 to 14 days. The arthritis is nonerosive. Joint problems precede the diagnosis of TAO by about 10 yrs on average. 

## 6. Laboratory Tests and Imaging 

There are no specific laboratory tests to aid in the diagnosis of TAO. A complete serologic proile to exclude other diseases that may mimic TAO should be obtained as in Table 3. A proximal source of emboli should be excluded with echocardiography (two-dimensional and/or transesophageal) and arteriography. Proximal arteries should show no evidence of atherosclerosis, aneurysm, or other source of proximal emboli. A pathologic specimen is needed to diagnose Buerger's disease in cases of proximal artery involvement or in unusual locations.

## 7. Differential Diagnosis 

Differential diagnosis of TAO includes atherosclerosis, emboli, autoimmune diseases scleroderma or the CREST syndrome, systemic lupus erythematosus, rheumatoid arthritis, mixed connective tissue disease, antiphospholipid antibody syndrome, and other types of vasculitis. In the presence of lower extremity involvement, the possibility of popliteal artery entrapment syndrome or cystic adventitial disease should be considered, both of which should be readily apparent on arteriography, computed tomography, or magnetic resonance imaging. If there is isolated involvement of the upper extremity, occupational hazards such as use of vibratory tools and hypothenar hammer syndrome should be considered. Two diagnostic criteria have been proposed by Shionoya and Olin as described in Table 4.

## 8. Treatment 

The most effective treatment for Buerger's disease is smoking cessation. All possible means should be used to encourage patients to give up the use of tobacco, in all its forms. Patient education is important, but only 43–70% of cases manage to give up smoking [39]. Psychological help may be useful in certain cases, but patients should be reassured that if they manage to give up smoking completely, the disease will go into remission and amputation can be avoided. Selective cannabinoid receptor antagonists, such as rimonabant, have shown good results in helping patient quit smoking [39].

### 8.1. Medical Treatment of Intermittent Claudication

#### 8.1.1. Platelet Inhibitors

*Aspirin*. Aspirin is effective in preventing secondary events and should be considered in all patients with PAD. Aspirin is not currently indicated, however, for the treatment of the symptoms of intermittent claudication.

*Clopidogrel*. Clopidogrel is an antiplatelet agent that has been shown to be more potent than aspirin in reducing secondary events in patients with atherosclerotic disease. There is no evidence, however, to suggest that the symptoms of claudication are reduced by long-term treatment with clopidogrel.

#### 8.1.2. Vasodilators

When vasodilator therapy is given, vessels proximal to the stenotic or occlusive lesion and vessels parallel to the lesion dilate and improve blood flow to that neighboring vascular bed. This improvement leads to a steal proximal to the stenotic or occlusive lesion, reducing blood flow from the already ischemic distal tissue. Vasodilators also have the capacity to reduce overall systemic vascular resistance, leading to a reduction in perfusion pressure. This reduction in perfusion pressure in conjunction with the steal phenomenon increases the ischemic insult to the underperfused extremity. This concept of enhancing blood flow by giving vasodilators systemically is probably incorrect.

A dihydropyridine calcium channel blocker, such as amlodipine or nifedipine, seems to be effective if vasospasm is present. In a study by Bagger et al. [42] increasing doses of verapamil was used in 44 patient of TAO; it was seen that there was an increased mean pain-free walking distance by 29% from 44.9 to 57.8 meters. There was no change in ankle/brachial index, suggesting that it was not purely secondary to blood flow. A theory that has evolved from this study is that the calcium channel blocker has a secondary effect—that of changing the oxygen extraction/utilization capacity. Calcium channel blockers may improve the efficiency of oxygen use in the extremity. A dose of verapamil up to 480 mg/day can be given as an adjuvant therapy to patients. 

*Pentoxyfylline*. Pentoxyfylline (Trental) is a methylxanthine derivative that has numerous effects. Its primary effect was thought to be an improvement in red blood cell deformability. Other effects include a decrease in blood viscosity, platelet aggregation inhibition, and a reduction in fibrinogen levels. Though usage of pentoxyfylline may increase the pain-free walking distance in many, the long-term benefit and improvement in quality of life is limited. 

*Cilostazol*. Cilostazol (Pletal) is a phosphodiesterase type III inhibitor which inhibits cyclic adenosine monophosphate (cAMP) phosphodiesterase. By increasing the levels of cAMP in platelets and blood vessels, there is inhibition of platelet aggregation and a promotion of smooth muscle cell relaxation. Numerous side effects occur with the long-term use of cilostazol the most common side effect is headache. Headache is probably secondary to the drug's vasodilatory properties. Patient have to be informed beforehand and possibly starting with a lower dose, such as 50 mg once a day, then after approximately 1 week increasing to 50 mg twice a day and then increasing to the recommended dosage of 100 mg twice a day may alleviate most of these headaches. Gastrointestinal side effects like diarrhea and bulky stools are also common. Another side effect is palpitations, and patients on long-term treatment must be evaluated for cardiovascular status and drug discontinued if patient develops congestive heart failure. Other drugs that have been proven beneficial in TAO patients with intermittent claudication are naftidrofuryl (Praxilene), levocarnitine, arginine, buflomedil, ketanserin, niacin, and lovastatin.

Surgical revascularization is rarely possible for patients with Buerger's disease due to the diffused vascular damage and the distal nature of the disease. Sasajima et al. [46] reported a five-year rate of primary patency of 49% and a secondary patency rate of 62% in 61 patients following infrainguinal bypass. The patency rates were 67% in those who discontinued smoking and 35% in those who continued to smoke. *In situ* bypass should be considered in patients with severe ischemia who have target vessels [47].

Sympathectomy may be performed to decrease arterial spasm in patients with Buerger's disease. A laparoscopic method for sympathectomy has also been used [48]. Sympathectomy has been shown to provide short-term pain relief and to promote ulcer healing in some patients with Buerger's disease, but no long-term benefit has been confirmed [49]. 

Omentopexy is an attractive option, but it needs proper mobilization of omentum by experts and more surgical time, increasing complications. Prolonged ileus, wound infection, closure difficulties, and hernia have been reported [50].

Ilizavor's technique is very effective to induce neoangiogenesis in TAO [51]. According to Ilizarov, gradual traction on living tissues can stimulate and maintain regeneration and active growth of tissues (bone, muscle, fascia, nerve, vessels, skin, and its appendages). This is called the “law of tension stress”. In a study by Patwa and Krishnan [52] who used Ilizaro's technique for TAO patient, a vertical tibial osteotomy with horizontal distraction was performed. It was seen that in 60 patients followed up for 5 yrs there was significant improvement in 53 patient in terms of ulcer healing, decrease in major amputation, rest pain, and claudication distance. Ilizavor's method is an excellent and cheap procedure in treatment of Buerger's disease. 

Spinal cord stimulators (SCS) are used extensively in refractory peripheral atherosclerotic disease. SCS may modulate painful stimuli through several mechanisms. Inhibition of sympathetic vasoconstriction improves the peripheral microcirculation. Nitric oxide and *γ*-aminobutyric acid systems in the spinal cord may be important intermediaries in SCS-induced pain relief [53]. Initial studies mainly aimed at pain relief in severe TAO. A study by Donas et al. in TAO patients, it was seen that regional perfusion index improved significantly after SCS though patient continued to smoke. It was shown that it not only helps relieve pain but also has a role in increasing peripheral microcirculation, thus, increasing limb survival, healing of trophic ulcers, and avoidance of amputation [54]. A study by Fabregat et al. [55] concluded that SCS should not only be considered as a last resort strategy for pain control, but also as a valid therapeutic option to improve perfusion of the limbs in the initial stages of the disease. Further large scale RCT are required to document advantage of SCS in early stages of TAO.

Prostacyclin derivatives have been evaluated in several studies and have been shown to be more effective than placebo in Buerger's disease. It is known that prostaglandin analogues facilitate relaxation of vascular smooth cells, inhibit platelet aggregation, and inhibit chemotaxis and cell proliferation. In a randomized study, 152 patients with Buerger's disease presenting with rest pain, with or without trophic changes, received intravenous iloprost or placebo. After 21–28 days of perfusion, the trophic lesions had healed or the pain had disappeared in 85% of the patients on iloprost and 17% of the patients on aspirin. At 6 months, amputation was required in 18% of the patients in the aspirin group and only 6% of the patients in the iloprost group [56]. Results of randomized trials comparing oral prostacyclin derivatives with placebo in peripheral arterial disease have been less impressive. The European thromboangiitis obliterans (Buerger's disease) study shows no significant difference was found between groups for the total healing of lesions [57]. Mohler et al. reported another double-blinded, randomized, controlled trial using beraprost sodium, an orally active prostaglandin I2 analogue for the treatment of intermittent claudication. Beraprost did not improve symptoms of intermittent claudication in patients with peripheral arterial disease [58]. 

This difference in effectiveness of oral and intravenous prostaglandin may be partially attributed to the fact that hospitalized patient adhere more to cessation of smoking as compared to home-based oral therapy which infact may change the progression of disease. A recent study by Bozkurt et al. [59] shows that in intravenous iloprost at dose 1 ng/kg/min the complete healing rate without pain or major amputation was 60.23% at 24 weeks. It is clearly evident that iloprost, when used intravenously, alleviates rest pain, improves ulcer healing, and decreases the rate of amputation in Buerger's disease.

There have been studies on role of endothelin 1 in pathogenesis of TAO [60]. It is shown that patients with TAO have elevated serum levels of endothelin 1. In a study by De Haro et al. 13 patients received oral endothelin antagonist bosentan at dose 65 mg twice daily for a month followed by 125 mg twice daily. It was seen that though patients continued to smoke, an overall 92% of patients showed clinical improvement with only 2 patients requiring minor amputations. Ten out of twelve patients showed an increase in distal blood flow demonstrated by digital angiography. Bosentan treatment may result in an improvement of clinical, angiographic, and endothelial function outcomes. Bosentan should be investigated further in the management of TAO patients [61].

There have been studies reporting use of gene transfer to induce therapeutic angiogenesis in TAO. Isner et al. [62] showed encouraging results in patients receiving intramuscular injections of vascular endothelial growth factor (VEGF). In a study by Kim et al. [63] he evaluated the safety of intramuscular VEGF gene transfer, using naked plasmid DNA in seven patients with TAO. The injections were well tolerated. Ischaemic ulcers healed or improved in four of six patients. Five of seven patients showed an increase in collateral vessels around the injection sites. 

Endothelial progenitor cells (EPCs) belong to an immature cell population that is capable of differentiating into mature endothelial cells. In adults, EPCs mainly reside in bone marrow (BM) and are more proliferative and migrative than terminally differentiated endothelial cells. EPCs can be clinically isolated as CD34*þ* or AC133*þ* mononuclear cells (MNCs) from adult BM or peripheral blood (PB) [64]. Tissue ischemia or systemic administration of G-CSF, GM-CSF, vascular endothelial growth factor, or estrogen enhances mobilization of EPCs from BM into PB, and the mobilized EPCs specifically home to sites of nascent neovascularization, thereby contributing to vascular repair.

Many studies have shown efficiency of autologous bone marrow cell injections into ischemic limbs [65]. In recent times, there is a trend towards usage of peripheral blood stem cells as alternate to BM stem cells. In a study by Moriya et al. [66] in 42 patients of TAO treated with peripheral blood mononuclear stem cells, improvement of ischemic symptoms was observed in 60% to 70% of the patients. The annual rate of major amputation was decreased markedly by treatment. 

In a study by Kawamoto et al. [67] in 17 patients with TAO who were treated with intramuscular injection of G CSF mobilized CD34^+^ cells from peripheral blood. During the 12-week observation after cell therapy, the Wong-Baker FACES pain rating scale, transcutaneous partial oxygen pressure, total or pain-free walking distance, and ulcer size serially improved in all patients. 

Cell therapies using bone marrow mononuclear cells (BM-MNCs) and peripheral blood mononuclear cells (PBMNCs) have effective outcomes in patients with peripheral artery disease and TAO. The adipose tissue is abundant in the human body and is consistently replenished. Therefore, this tissue is an ideal source of MSCs. It has been shown that adipose tissue derived MSCs (ATMSCs) have characteristics similar to those of bone marrow stromal cells (BMSCs) [68]. ATMSCs differentiate into endothelial cells and have a proangiogenic effect. In a study by Lee et al. [69] 15 patients with TAO were treated with intramuscular injections of ATMSCs; it was seen that clinical improvement occurred in 66.7% of patients. Five patients required minor amputation during followup, and all amputation sites healed completely. At 6 months, significant improvement was noted on pain rating scales and in claudication walking distance. Digital subtraction angiography before and 6 months after ATMSCs implantation showed formation of numerous vascular collateral networks across affected arteries. Further large-scale randomized trials are required to assess the long-term benefits of stem cell therapy.

## 9. Conclusion

TAO is a distinct form of systemic vasculitis of unknown etiology though strongly linked to cigarette smoking. Clinical features and angiography form the main basis of diagnosis. Abstinence from smoking is the only definitive treatment to prevent disease progression. Medical line of treatment with vasodilators, pentoxyfylline, and cilostazol may help improve pain-free walking distance but cannot prevent disease progression. Surgical treatment in form of revascularization, sympathectomy, Ilizarov, and omentopexy increases peripheral blood flow and decreases the rate of amputations. Newer therapy with prostaglandins, bosentan, and stem cell therapy has shown promising results. Early diagnosis and aggressive therapy can decrease patient symptoms and chances of major amputations.

## Figures and Tables

**Table 1:** Rutherford classification.

Grade	Category	Clinical
0	0	Asymptomatic
I	1	Mild claudication
I	2	Moderate claudication
I	3	Severe claudication
II	4	Rest pain
III	5	Ischemic ulcer not exceeding digits
IV	6	Severe ischemic ulcer or gangrene

*Adapted from [40].

**Table 2:** Leriche-fontaine classification.

Stages	Symptoms	Pathophysiology	Pathophysiological classification
I	Asymptomatic or effort pain.	Relative hypoxia	Silent arteriopathy
II A	Effort pain/pain-free walking distance >200 m.	Relative hypoxia	Stabilized arteriopathy, noninvalidant claudication
II B	Pain-free walking distance <200 m.	Relative hypoxia	Instable arteriopathy, invalidant claudication
III A	Rest pain, ankle arterial pressure >50 mm Hg.	Cutaneous hypoxia, tissue acidosis, ischemic neuritis	Instable arteriopathy, invalidant claudication
III B	Rest pain, ankle arterial pressure <50 mm Hg.	Cutaneous hypoxia, tissue acidosis, ischemic neuritis	Instable arteriopathy, invalidant claudication
IV	Trophic lesions, necrosis or gangrene.	Cutaneous hypoxia, tissue acidosis, necrosis	Evolutive arteriopathy

*Adapted from [41].

**Table 3:** Diagnostic investigation for Buerger's disease.

Blood count	
Liver function	
Renal function	
Fasting blood sugar	
Erythrocyte sedimentation rate	
C-reactive protein	
Antinuclear antibodies	
Rheumatoid factor	
Complementary measurements	
Anticentromere antibodies (for CREST)	
Anti-Scl-70 antibodies (for scleroderma)	
Antiphospholipid antibodies	
Lipid profile	
Urinalysis	
Toxicology screen for cocaine and cannabis	
Cryoproteins	
Segmental arterial Doppler pressures	
Arteriography	
Echocardiography (to exclude source of emboli)	
Computed tomography (to exclude potential source of emboli)	
Biopsy (In proximal artery involvement or unusual locations)	
Complete thrombophilia screen: proteins G and S, antithrombin III, factor V Leiden, prothrombin 20210, and homocysteinemia	
Hand radiographs (to exclude calcinosis)	

CREST Calcinosis, Raynaud's phenomenon, esophageal dysmotility, sclerodactyly, and telangiectasia. *Adapted from [43].

**Table 4:** Diagnostic criteria.

SHIONOYA criteria [44]	OLIN criteria [45]
Onset before age 50	Onset before age 45
Smoking history	Current (or recent past) tobacco use
Infrapopliteal arterial occlusions upper limb involvement or phlebitis migrans	Distal extremity ischemia (infrapopliteal and/or intrabrachial), such as claudication, rest pain, ischemic ulcers, and gangrene documented with noninvasive testing
Absence of atherosclerotic risk factors other than smoking	Laboratory tests to exclude autoimmune or connective tissue diseases and diabetes mellitus
	Exclude a proximal source of emboli with echocardiography and arteriography
	Demonstrate consistent arteriographic findings in the involved and clinically noninvolved limbs

A biopsy is rarely needed to make the diagnosis unless the patient presents with an unusual characteristic, such as large artery involvement or age greater than 45 years.
